# Supplementary material for: A bacterial membrane sculpting protein with BAR domain-like activity
Source: eLife. 2021 Oct 13;10:e60049. doi: 10.7554/eLife.60049 (PMC8687657; doi:10.7554/eLife.60049)
Supplement: Supplementary file 2. [file elife-60049-supp2.docx]

Supplemental Dataset 1. Multiple protein alignment of the five *Shewanella* spp. homologs of BdpA, and 61 most diverse representative sequences obtained from CDD.

cov pid  **1** **[ . . . . : . . . . 1** **100**

1 BdpA_Shewanella_genus 100.0% 100.0% **----------------------------------------------------------------------------------------------------**

2 BdpA_Shewanella_oneidensis 100.0% 96.0% **----------------------------------------------------------------------------------------------------**

3 BdpA_Shewanella_xiamenensis 100.0% 96.6% **----------------------------------------------------------------------------------------------------**

4 BdpA_Shewanella_decolorationis_S12 100.0% 97.2% **----------------------------------------------------------------------------------------------------**

5 PmtA_Agrobacterium_tumefaciens 77.8% 4.8% **----------------------------------------------------------------------------------------------------**

6 FtsA_Shewanella_oneidensis 98.9% 4.3% **------------------------------------------DLDSIVRSVQRALDQAELMADCQVSSVYLSIS-GKHIACQNENGMVSINDEEVTQEDV**

7 FtsA_Escherichia_coli 100.0% 3.3% **------------------------------------------DLESVVKCVQRAIDQAELMADCQISSVYLALS-GKHISCQNEIGMVPISEEEVTQEDV**

8 MamY_Magnetospirillum_magneticum 85.2% 8.8% **----------------------------------------------------------------------------------------------------**

9 gi99031622pdb1X03A 80.7% 6.9% **-----------------------------------------------------------KVDVTSRA--------VXEIXTKTIEYLQPNPASRAKLSXI**

10 gi149241632pdb2ELBA 80.7% 4.8% **-----------------------------------------------------------DATAISNY--------XNQLYQAXHRIYDAQNELSAAT---**

11 gi151568102pdb2V0OA 55.1% 4.0% **GFDVLYHNMKHGQISTKELADFVRERATIEEAYSRSMTKLAKSASNYSQLGTFAPVWDVFKTSTEKLANCH----LDLVRKLQELIKEVQKYGEEQVKSH**

12 gi119389482pdb2FICA 84.1% 7.1% **-----------------------------------------------------------NFNKQLTE--------GTRLQKDLRTYLASVKAMHEA----**

13 gi14277759pdb1I49A 83.5% 7.8% **--------------------------------------------------------------------------LLRETKRKYESVLQLGRALTAHL---**

14 gi116666967pdb2D1LA 80.1% 3.8% **-------------------------------------------------------------TIISDXKGSY--PVWEDFINKAGKLQSQLRTTVVAA---**

15 gi73536002pdb1ZWWA 80.7% 6.4% **-----------------------------------------------------------KVDVTSRA--------VMEIMTKTIEYLQPNPASRAKLSMI**

16 gi162330107pdb2RAIA 75.6% 6.8% **----------------------------------------------------------------------------------------------------**

17 gi56404535spQ6XZF7.1DNMBP_HUMAN 80.7% 5.6% **-----------------------------------------------------------NFRMQERL--------IKSFIRDLSLYLQHIRESACV----**

18 gi41018158spQ9Y371.1SHLB1_HUMAN 85.2% 5.3% **-----------------------------------------------------------KAECTKIW--------TEKIMKQTEVLLQPNPNARIEEFVY**

19 gi119584059gbEAW63655.1 85.2% 6.0% **-----------------------------------------------------------KLQQLEEQ--------TRRLQKDMKKSTDADLAMSKS----**

20 gi79512687refNP_196834.3 77.8% 4.3% **-----------------------------------------------------------SAEILRER--------SLKFYKGCRKYTEGLGEAYDGD---**

21 gi6322148refNP_012223.1 80.7% 8.1% **--------------------------------------------------------------SVNEF--------SRSVASKLTELTHATSASEAQNILV**

22 gi11359444pirT49496 77.8% 7.5% **------------------------------------------K--------------------------------TGNMRMQMKRLIKRAEQVYAAQ---**

23 gi223461365gbAAI40764.1 82.4% 3.5% **----------------------------------------------------------------------------------------------------**

24 gi71052100gbAAH51194.2 80.7% 4.5% **----------------------------------------------------------------------------------------------DVVEIE**

25 gi119370361spQ9ULH1.3ASAP1_HUMAN 83.5% 6.6% **----------------------------------------------------------------------------------------------ALDQDR**

26 gi148910033gbABR18100.1 64.2% 4.7% **-----------------------------------------------------------STRAAKHFQ--------RDIVRGVEGHISAGSKQMEYAIKL**

27 gi134128spP25343.1RV161_YEAST 86.9% 6.0% **-----------------------------------------------------------RYKVLQRA--------GEALQKEAKGFLDSLRAVTAS----**

28 gi190409230gbEDV12495.1 73.3% 8.4% **--------------------------------------------------------------RVDALEDWIEKTVDFFDQKYKVSFEDFRRAKETLLSQL**

29 gi123888297spQ1LU86.1F92A1_DANRE 81.8% 6.4% **----------------------------------------------------------------------------------------------------**

30 gi74676202spO94478.1MU137_SCHPO 83.0% 6.8% **------------------------------------------------EIAIREEGLSKLFQATTIW--------IDSILKKVDGEDKEKCLACEN----**

31 gi81880317spQ99N37.1RHG17_RAT 86.9% 8.4% **---------------------------------------------KTEVLSEDLLQIERRLDTVRSM--------CHHSHKRLIACFQGQHGTDAE----**

32 gi730455spP39743.1RV167_YEAST 88.1% 3.5% **-----------------------------------------------------------RFQELEQE--------TKKLSEESKRYSTAVNGMLTH----**

33 gi23943858refNP_037453.1 84.1% 6.1% **----------------------------------------------------------------------------------------------------**

34 gi226520535gbACO66524.1 91.5% 7.2% **---------------------------------------------GV----QRQELLLNKIKTDGKR------------------LAAAIQEQADA----**

35 gi66805481refXP_636473.1 80.1% 4.0% **-----------------------------------------------------------KLFTLFKL--------MKRLNKNVDKYEATLKEINILQ---**

36 gi66814788refXP_641573.1 86.9% 3.9% **------------------------------------------------------------AMENKKT--------LKKIIKSSKHWSESVKAYCTT----**

37 gi66811612refXP_639985.1 82.4% 4.8% **-----------------------------------------------------------RTFSTEER--------LVKIQKNSKKLIELYKDLNKIT---**

38 gi66808477refXP_637961.1 83.5% 4.9% **-----------------------------------------------------------KLRVIKTE--------YSQIYTVGKLYAQETEKSTQQGS--**

39 gi66815771refXP_641902.1 84.1% 8.3% **-----------------------------------------------------------KTTETKDF--------LRKLTKSVEKETLSSGVSIQDGT--**

40 gi226457821gbEEH55119.1 89.8% 6.5% **---------------------------------------------RV----QRQEALLNLIQKDGKARPCSHWSPYDRVRVVNAAYAKAIDAMHAA----**

41 gi66826123refXP_646416.1 75.0% 4.2% **------------------------------------------------------------ALQNFKKT-------CTKIIKSSKKYYEVEPDSFKEQGH-**

42 gi66815801refXP_641917.1 86.9% 4.5% **----------------------------------------------------------------------------------------------------**

43 gi115458110refNP_001052655.1 86.4% 6.8% **----------------------------------------------------------------------------------------------------**

44 gi218202434gbEEC84861.1 86.4% 3.1% **----------------------------------------------------------------------------------------------------**

45 gi67468783refXP_650391.1 81.8% 6.1% **--------------------------------------------------------------ELQNT--------IKKLKKASSSYSKLPDAGRSST---**

46 gi67467978refXP_650059.1 90.3% 7.8% **---------------------------------------------SIDTDFKEVHK---KTKLHYKL--------LIQTLLTLRHYNESQKKMLHKK---**

47 gi67468969refXP_650476.1 79.5% 4.7% **-----------------------------------------------------------QLFLKEKK--------CRHFLTKVRKICQYYFCENKQT---**

48 gi183234774refXP_656019.2 83.0% 5.2% **-----------------------------------------------------------KLSESYKK--------FKNILDVIKKLAPTVHATNLMQ---**

49 gi30685727refNP_180907.2 86.4% 5.2% **----------------------------------------------------------------------------------------------------**

50 gi30688552refNP_189326.2 84.7% 7.9% **----------------------------------------------------------------------------------------------------**

51 gi67471335refXP_651619.1 82.4% 6.5% **-----------------------------------------------------------EIIEKEKK--------MKEIIERVSKITTSAEKLIRIP---**

52 gi66809051refXP_638248.1 81.8% 4.9% **---------------------------------------------------------------NFNY--------LKDIHTKINKRSQSSQVCSREGS--**

53 gi183233566refXP_655372.2 83.5% 6.2% **-----------------------------------------------------------KLSDMSNE--------FQKVITYVEELPKTVSNISGNH---**

54 gi118394946refXP_001029831.1 74.4% 6.6% **----------------------------------------------------------------------------------------RAENM-------**

55 gi195998291refXP_002109014.1 76.7% 4.5% **------------------------------------------D--------------------------------VLNLQQALTSYSQILTKMSNAG---**

56 gi67480227refXP_655464.1 79.0% 5.5% **-----------------------------------------------------------RVIQNKKL--------YKQI----------LHCAEILP---**

57 gi123437348refXP_001309471.1 82.4% 4.9% **----------------------------------------------------------------------------------------------------**

58 gi66817346refXP_642526.1 77.8% 3.0% **-------------------------------------------------------------------NLNDIKVLLNDIIKSCKNYYDRGEQFAKTQVKW**

59 gi66811120refXP_639268.1 84.1% 5.0% **----------------------------------------------------------------------------------------------------**

60 gi183231363refXP_656153.2 81.2% 4.2% **-------------------------------------------DLKGKPKDVVCEQIEKDKKDLLAL--------SAKIKKAQEKFEKLGKDAKEQY---**

61 gi159480288refXP_001698216.1 86.4% 5.0% **----------------------------------------------------------------------------------------DLRSMHKEIDAK**

62 gi67476764refXP_653934.1 88.6% 9.5% **-----------------------------------------------------------KFKYQEVG--------LNRLYHLIANNLDLMIQVQHTR---**

63 gi219120544refXP_002181008.1 89.8% 13.5% **---------------------------------------------RYDTWCKQVRGLIVALQQHHAV--------MGQIEKTRANLSKHFAALSVKTPI-**

64 gi154416821refXP_001581432.1 76.1% 4.8% **----------------------------------------------------------------------------------------------------**

65 gi167376424refXP_001733989.1 90.3% 8.3% **---------------------------------------------SIDTDFKEIHK---KTKLHYKL--------LIQTLLMLRHYNESQKKMLNTK---**

66 gi123447384refXP_001312432.1 80.1% 7.4% **--------------------------------------------------------------------------NFSILQEDTSQIITTLAQLQQQIS--**

67 gi168036718refXP_001770853.1 79.0% 4.5% **----------------------------------------------------------------------------------------------------**

68 gi123475188refXP_001320773.1 80.1% 7.0% **-------------------------------------------------------------------DYTQAKEAFKTICLDTVQIIGCIIQMSNQMQ--**

69 gi167538764refXP_001751041.1 89.2% 8.3% **-----------------------------------------------------------SEVVTDRY--------NNTVSKACDAMVEKGFAYERSV---**

cov pid **101**  **. . . . : . . . . 2** **200**

1 BdpA_Shewanella_genus 100.0% 100.0% **-------------------------ATATLNVVS--AGAYSAF-------AEPF------------KSFLAATFDRS-NYDNSDLSRSDKK---------**

2 BdpA_Shewanella_oneidensis 100.0% 96.0% **-------------------------ATATLNVVS--AGAYSAF-------AEPF------------KSFLAATFDRS-NYDNSDLSRSDKK---------**

3 BdpA_Shewanella_xiamenensis 100.0% 96.6% **-------------------------ATATLNVVS--AGAYSAF-------AEPF------------KSFLAATFDRS-NYDNSDLSRSDKK---------**

4 BdpA_Shewanella_decolorationis_S12 100.0% 97.2% **-------------------------ATATLNVVS--AGAYSAF-------AEPF------------KSFLAATFDRS-NYDNSDLSRSDKK---------**

5 PmtA_Agrobacterium_tumefaciens 77.8% 4.8% **------------------------------------------------------------------------MALNLKQRLEQKFEEEIRFFKGMV--SQ**

6 FtsA_Shewanella_oneidensis 98.9% 4.3% **------DNVIHTARSVKIPTERRILHVLPQEYAIDVQDGIRSPIGMSGMRMEAKVHIVTCAN--DMAKNITKSVERC-GLKVDDLVFSGIASADAVLTFD**

7 FtsA_Escherichia_coli 100.0% 3.3% **------ENVVHTAKSVRVRDEHRVLHVIPQEYAIDYQEGIKNPVGLSGVRMQAKVHLITCHN--DMAKNIVKAVERC-GLKVDQLIFAGLASSYSVLTED**

8 MamY_Magnetospirillum_magneticum 85.2% 8.8% **--------------------------------------------------------------------FNKAAFGKLNSASRAALIGAVIWAVLSI----**

9 gi99031622pdb1X03A 80.7% 6.9% **------NTXSKIRGQEKGPGYPQAEALLAEAXLK--FGRELGD----DCNFGPA------------LGEVGEAXREL-SEVKDSLD--------------**

10 gi149241632pdb2ELBA 80.7% 4.8% **-------------------------HLTSKLLKE--YEKQRFPLG------GDDEVX------SSTLQQFSKVIDEL-SSCHAVLSTQLADA--------**

11 gi151568102pdb2V0OA 55.1% 4.0% **---------------------------------------------------------------KKTKEEVAGTLEAV-----------------------**

12 gi119389482pdb2FICA 84.1% 7.1% **------------------------SKKLNECLQE--VYEPDWP----GRDEANK-------------------IAENNDLLWMDYH--------------**

13 gi14277759pdb1I49A 83.5% 7.8% **-------------------------YSLLQTQHA--LGDAFADLSQKSPELQEE------------FGYNAETQKLL-CKNGETLLGAVNFF--------**

14 gi116666967pdb2D1LA 80.1% 3.8% **-------------------------AAFLDAFQK--VADXATNTRG-------------------GTREIGSALTRX-CXRHRSIEAKLRQFSS------**

15 gi73536002pdb1ZWWA 80.7% 6.4% **------NTMSKIRGQEKGPGYPQAEALLAEAMLK--FGRELGD----DCNFGPA------------LGEVGEAMREL-SEVKDSLD--------------**

16 gi162330107pdb2RAIA 75.6% 6.8% **---------------------------------------------------------------------FTKAXDDGVKELLTVGQEHWKRCTG-PLPKE**

17 gi56404535spQ6XZF7.1DNMBP_HUMAN 80.7% 5.6% **------------------------KVVAAVSMWD--VCMERGH----RD-----------------LEQFERVHRYISDQLFTNFK--------------**

18 gi41018158spQ9Y371.1SHLB1_HUMAN 85.2% 5.3% **------EKLDR-----KAPSRINNPELLGQYMID--AGTEFGP----GTAYGNA------------LIKCGETQKRI-GTADRELI--------------**

19 gi119584059gbEAW63655.1 85.2% 6.0% **------------------------AVKISLDLLSNPLCEQDQD----LLNMVTA---------------LDTAMKRM--DAFNQEK--------------**

20 gi79512687refNP_196834.3 77.8% 4.3% **-------------------------IAFASALET--FGG-----G------HNDPISV--AFGGPVMTKFTIALREI-GTYKEVLRSQVEHI--------**

21 gi6322148refNP_012223.1 80.7% 8.1% **------APGPIKEPKTLNYALSKVALNSSECLNK----MFPTE----EQPLASA------------LLQFSDVQAKI-AQARIQQD--------------**

22 gi11359444pirT49496 77.8% 7.5% **-------------------------TEANDAFLQ----------------------------------FMEA--LRD---VSSTNANAVQPAIE------**

23 gi223461365gbAAI40764.1 82.4% 3.5% **-------ELERTNKFIKDIIKDGNALISAMRNYSSAVQKFSQTLQSFQFDFIGDTLTDDEIN-------IAESFKEF-AELLNEVENERMMMVH------**

24 gi71052100gbAAH51194.2 80.7% 4.5% **------AKLDKLVKLCSGMVEAGKAYVSTSRLFVSGVRDLSQQCQ-------GDTV-------------ISECLQRF-ADSLQEVVNYHMNLFD------**

25 gi119370361spQ9ULH1.3ASAP1_HUMAN 83.5% 6.6% **------TALQKVKKSVKAIYNSGQDHVQNEENYAQVLDKFGSNFLS-----------RDNPD-------LGTAFVKF-STLTKELSTLLKNLLQ------**

26 gi148910033gbABR18100.1 64.2% 4.7% **ADDCC---------------------------------KYANDGPNSNSALSRASFYFGSSH--NAMEKEREDLHRI----------------FGV----**

27 gi134128spP25343.1RV161_YEAST 86.9% 6.0% **------------------------QTTIAEVISN--LYDDSKY----VAGGGYN----------VGNYYLQCVQDFD-SETVKQLD--------------**

28 gi190409230gbEDV12495.1 73.3% 8.4% **LPPP---------------------ALLSNGFVSN------------------------QSFTPRLIDSFNKDYYDF-SMKLLQIVKGDDSSHSTAL---**

29 gi123888297spQ1LU86.1F92A1_DANRE 81.8% 6.4% **------------NITSVEKHFGDLCQLFAAYVRKTARLRDKADLLVKEINVYADTETP------N----LKCGLKNF-ADQLAKVQDYRQAEVE------**

30 gi74676202spO94478.1MU137_SCHPO 83.0% 6.8% **---------------------------LGKVMIN--HSKELPQ----DSSYGIT------------LSQLGKANLKI-GEHQTSLA--------------**

31 gi81880317spQ99N37.1RHG17_RAT 86.9% 8.4% **------------RRHKKLPL-----TALAQNMQE--ASAQL-E----ESLLGKM------------LETCGDAENQL-AFELSQHE--------------**

32 gi730455spP39743.1RV167_YEAST 88.1% 3.5% **------------------------QIGFAKSMEE--IFKPISG----KMSDPNATIPEDNPQGIEASEQYRAIVAEL-QETLKPDL--------------**

33 gi23943858refNP_037453.1 84.1% 6.1% **----------------------------------------------------------QFAISRELIRNIYNSFHKLRDRAERIASRAIDNAADLLIFGK**

34 gi226520535gbACO66524.1 91.5% 7.2% **------------------------AKSMASHIAS--LAETSIE----L-----------GE--GPEADARRAVVERA-RQLMEVMDVMDQD---------**

35 gi66805481refXP_636473.1 80.1% 4.0% **----------------------NEISHDLLALNE--NDPAI----K------------------AY----QEVSTSL-EQERIRIEE-------------**

36 gi66814788refXP_641573.1 86.9% 3.9% **------------------------ASQFSEELLK--HSEQLSPSPKKETHLNVA------------LLSFGHTLKSV-NSINDQML--------------**

37 gi66811612refXP_639985.1 82.4% 4.8% **----------------------TDIAQDACDLYD--TQDPMWTPGS------------------KL----RDVATNN-DKHLLDYTE-------------**

38 gi66808477refXP_637961.1 83.5% 4.9% **--------------Q------------FADALAQ--FGTGFV----SNEQVSEA------------LKNVGIQLKSV-EQARQSCNVNSVQS--------**

39 gi66815771refXP_641902.1 84.1% 8.3% **--------------E------------LADNFLD--YSVHVRDNQSDLVILSGI------------LSKIGEFQAGF-EDLKSKLNSSLIND--------**

40 gi226457821gbEEH55119.1 89.8% 6.5% **------------------------ARTMAGHIAE--LTEMDPD----EPLPDPGPISPGGT--GPVS--REAVVDRA-ARLTEVMDALEND---------**

41 gi66826123refXP_646416.1 75.0% 4.2% **---------------------------FSEHLINV--------KACFDGNIDPALAA--------SIDQFSSSIKKI-GYLRSELSTRTHTSFA------**

42 gi66815801refXP_641917.1 86.9% 4.5% **------------YTRTLNLKLTKHSKTNQLLIEENKQLSEQI-LLYSNLFINNEESSL------NVCEPLSNVFKIV-GEMINEIENYRQTFEQ------**

43 gi115458110refNP_001052655.1 86.4% 6.8% **-----------------------------------------------------------------EMEEMRNCYDSLLSAAAATMNSVYEFA------EA**

44 gi218202434gbEEC84861.1 86.4% 3.1% **-----------------------------------------------------------------DMADMRSCYDNLLSVAAAIANSAYEFS------EA**

45 gi67468783refXP_650391.1 81.8% 6.1% **----------------------VCHMQAMKDGKD--LLG-EGCCCQ------------------EC----YSIFEEI-DMKGHLYGM-------------**

46 gi67467978refXP_650059.1 90.3% 7.8% **-------------------------VELSKALIE--MSKEDIQ-------LNSI------------ATKYMTTCECL-ELGLTTCLTSMED---------**

47 gi67468969refXP_650476.1 79.5% 4.7% **----------------------EKIFCSLRKIYV--EGKNEVNSLT------------------NTLNSLQKTFQLN-KKEITKMEK-------------**

48 gi183234774refXP_656019.2 83.0% 5.2% **----------------------VEVLTSLGDCV----VNTSPETKS------------------DI-DSIISTFQKI-DEGVNTYET-------------**

49 gi30685727refNP_180907.2 86.4% 5.2% **-----------------------------------------------------------------DVEDMRDCYDSLLNAAAATANSAYEFS------ES**

50 gi30688552refNP_189326.2 84.7% 7.9% **-----------------------------------------------------------------DMQDMRECYDRLLAAAAATANSAYEFS------ES**

51 gi67471335refXP_651619.1 82.4% 6.5% **----------------------KEVFLILHDCS----GEKS-KAKE------------------ES-ERMINVFSSM-EEGGKHIKE-------------**

52 gi66809051refXP_638248.1 81.8% 4.9% **--------------S------------LAETFKN--YGTMLI-SQSDNV-MGQC------------MFKVGDFQKEY-EEIRDQLDIQSMSG--------**

53 gi183233566refXP_655372.2 83.5% 6.2% **----------------------LNILNSLHLCVQ--ISEGEEAKME------------------QI-SDTISIFQKM-ERDAARY-Q-------------**

54 gi118394946refXP_001029831.1 74.4% 6.6% **-KAFLKKFAKNLKDLVKNGEIYSQTLKALSK----EVSL---NLNLVEDNKELFSMFTYTS----------EYFKDL-----TNFYDALNFN--------**

55 gi195998291refXP_002109014.1 76.7% 4.5% **-------------------------LLLAEAFTS----------------------------------VFKETPLWE---ATFKHTNTMNE-IK------**

56 gi67480227refXP_655464.1 79.0% 5.5% **----------------------TQAFTPI-DTL----KQTA--IMV------------------ND-SRIVDILNSI-EKTGF---E-------------**

57 gi123437348refXP_001309471.1 82.4% 4.9% **-------RL-------------DSIQKMASLIIE------------AERTFGKYVEVG---------KQLCGIMHKL-SVSFTNFGNIEGDPNI------**

58 gi66817346refXP_642526.1 77.8% 3.0% **SQSFSKDYEKSTTHSSSAQQVYQQQQQAAAANGNGETSTSDSNLGFIRALEQFTSSITKTS----------GFESEW-----IN-------S--------**

59 gi66811120refXP_639268.1 84.1% 5.0% **--------------------LYDETIRLSKKMVSKTQKYITSSTPLQESLQELANYYTNEHFTGRSIENILKIHFEL-----AAFREKQQTS--------**

60 gi183231363refXP_656153.2 81.2% 4.2% **----------------------DEHKKALKLCAD--VVTCDPNPFN------------------ES----IKIIEEN-DEKAKKYEE-------------**

61 gi159480288refXP_001698216.1 86.4% 5.0% **-------------------------FANLRAILSSPLPRTYEEGANGVVPVSEEAKLIGQGI---AVDRLQESANEL-----------------------**

62 gi67476764refXP_653934.1 88.6% 9.5% **-------------------------IDITDALVQ--LTDPIVE-------INSA------------ALKYKTTCEIE-SMAVSKYKQTVQK---------**

63 gi219120544refXP_002181008.1 89.8% 13.5% **-------------------------HEATGMLPS--ADRPSST-------VNSY------------ASIHDTLSAKT-QSYVAKYQQFVID---------**

64 gi154416821refXP_001581432.1 76.1% 4.8% **-------RF-------------RNIMQINKLMGI------------ITNHLDTYCQLG---------NKIGSTFEEI-ARCLSEMDIITHDDSY------**

65 gi167376424refXP_001733989.1 90.3% 8.3% **-------------------------VKLSNALIE--ISKEDIQ-------LNSI------------ATKYMTTCECL-ELGLTTCLTSIED---------**

66 gi123447384refXP_001312432.1 80.1% 7.4% **--------------N---------VSRICARLGSN-LTVWSEEFPENVKNEAIT------------IESFGKQFDNL----------TTNFFVP------**

67 gi168036718refXP_001770853.1 79.0% 4.5% **-----------------------------------------------------------VTNGMKDIRSIQKKYESLVSLSTEVSHRAYDLS------TA**

68 gi123475188refXP_001320773.1 80.1% 7.0% **--------------V---------LSRSVTKIGAD-LGISFITASQNSKGEAKA------------IEMFGKQLESI----------VSNQFAR------**

69 gi167538764refXP_001751041.1 89.2% 8.3% **-------------------------GAFVDELHA--LGNDA-AYR------NDATLP-------SALNQFTSALREL-QSHRTLLLQQAKAT--------**

cov pid **201**  **. . . . : . . . . 3** **300**

1 BdpA_Shewanella_genus 100.0% 100.0% **-------FAEE----------NGLKIYQQAERF------MAEIE----KELQQVT-ALDNDLVTMQKNLDTFRKDLDKHLRSYLQ-----HASIART--P**

2 BdpA_Shewanella_oneidensis 100.0% 96.0% **-------FAEE----------NGLKIYQQAERF------MAEIE----KELQQVT-ALDNDLVSMQKNLDNFRKDLDKHLRSYLQ-----HASIART--P**

3 BdpA_Shewanella_xiamenensis 100.0% 96.6% **-------FAEE----------NGLKIYQQAERF------MSEIE----KELQQVT-ALDNDLVSMQKNLDNFRKDLDKHLRSYLQ-----HASIART--P**

4 BdpA_Shewanella_decolorationis_S12 100.0% 97.2% **-------FAEE----------NGLKIYQQAERF------MSEIE----KELQQVT-ALDNDLVSMQKNLDAFRKDLDKHLRSYLQ-----HASIART--P**

5 PmtA_Agrobacterium_tumefaciens 77.8% 4.8% **PKKVGAIVPTSSIT---------AKKMASVINPHSG-LPVLELGPGTGVITKAIL--ARGIKPENLTAIEYSTDFYNQLLRSYPG----VNFINGDA---**

6 FtsA_Shewanella_oneidensis 98.9% 4.3% **EKDLGVCIVDIGGGTTDIAVYTNGALRHCAVVP------VAGNQVTN-DIAKIFR-TPSSHAEQIKVQFACARSSMVSREDSIEVPSVGGRPSRSMS--R**

7 FtsA_Escherichia_coli 100.0% 3.3% **ERELGVCVVDIGGGTMDIAVYTGGALRHTKVIP------YAGNVVTS-DIAYAFG-TPPSDAEAIKVRHGCALGSIVGKDESVEVPSVGGRPPRSLQ--R**

8 MamY_Magnetospirillum_magneticum 85.2% 8.8% **---VYLTIFNGWKNLFTMLPHEFFIVLLSIALPIG----LTVLILMLSRIVKSVD-TLKSEVTTLSRNDVSSEGSVAMLADLFREHRAAIAAQVEAQ-VE**

9 gi99031622pdb1X03A 80.7% 6.9% **----------------IE-------VKQNFIDP------LQNLHD---KDLREIQ-HHLKKLEGRRLDFDYKKKRQGKIP-------------------D**

10 gi149241632pdb2ELBA 80.7% 4.8% **-----------------------------XXFP------ITQFKERD---LKEIL-TLKEVFQIASNDHDAAINR-------YSR----LSKKREND--K**

11 gi151568102pdb2V0OA 55.1% 4.0% **------------------------QTIQSITQA------LQKSKENY--NAKCVEQERLKKEGATQREIEKAAVKSKKATDTYKLYV--EKYALAKADFE**

12 gi119389482pdb2FICA 84.1% 7.1% **----------------QK-------LVDQALLT------MDTYL----GQFPDIK-SRIAKRGRKLVDYDSARHHYESLQTAKKK---D-EAKIAKA--E**

13 gi14277759pdb1I49A 83.5% 7.8% **------------------------------VSS------INTLVTKT---MEDTL-MTVKQYEAARLEYDAYRTDLEELSLGPRD--AGTRGRLESA--Q**

14 gi116666967pdb2D1LA 80.1% 3.8% **------------------------ALIDCLINP------LQEQXEEWKKVANQLDKDHAKEYKKARQEIKKKSSDTLKLQKKAKKVDAQGRGDIQPQ-LD**

15 gi73536002pdb1ZWWA 80.7% 6.4% **----------------ME-------VKQNFIDP------LQNLHD---KDLREIQ-HHLKKLEGRRLDFGYKKKRQGKIP-------------------D**

16 gi162330107pdb2RAIA 75.6% 6.8% **YQKIGKALQSLATVFSSSGYQGETDLNDAITEAGKTYEEIASLVAEQ-----------------PKKDLHFLXECNHEYKGFLGC-FPDIIGTHKGA--I**

17 gi56404535spQ6XZF7.1DNMBP_HUMAN 80.7% 5.6% **----------------ER-------TERLVISP------LNQLL----SMFTGPH-KLVQKRFDKLLDFYNCTERAEKLKDK-------------KT--L**

18 gi41018158spQ9Y371.1SHLB1_HUMAN 85.2% 5.3% **----------------QT-------SALNFLTP------LRNFIE---GDYKTIA-KERKLLQNKRLDLDAAKTRLKKAKAAETR---------NSS--E**

19 gi119584059gbEAW63655.1 85.2% 6.0% **----------------VNQ------IQKTVIEP------LKKFG----SVFPSLN-MAVKRREQALQDYRRLQAKVEKYEEKEKTGPVL--AKLHQA--R**

20 gi79512687refNP_196834.3 77.8% 4.3% **-----------------------------LNDR------LLQFANMD---LHEVK-EARKRFDKASLTYDQAREK-------FLS----LRKGTKSD---**

21 gi6322148refNP_012223.1 80.7% 8.1% **----------------TL-------IQTKFNKN------LRERLSF---EIGKAD-KCRKDVHSMRLRYDVARTNLANNKKPEK---------------E**

22 gi11359444pirT49496 77.8% 7.5% **-HYFDKIAWEILRF----ERQNTQNLQKIVIDP------MNKLYQLD------IK-QAEAKKRDFEEESKDFYAYVSRYLGQRHD-----SVKAKQS--D**

23 gi223461365gbAAI40764.1 82.4% 3.5% **------------------------NASDLLIKP------LENFRKEQ---IGFTK-ERKKKFEKDGERFYSLLDRHLHLSSKKKE----------SQ--L**

24 gi71052100gbAAH51194.2 80.7% 4.5% **------------------------QAQRSVRQQ------LQSFVKED---VRKFK-ETKKQFDKVREDLELSLVRNAQ-APRHRP----------HE--V**

25 gi119370361spQ9ULH1.3ASAP1_HUMAN 83.5% 6.6% **------------------------GLSHNVIFT------LDSLLKGD---LKGVKGDLKKPFDKAWKDYETKFTKIEK---EKRE----------HA--K**

26 gi148910033gbABR18100.1 64.2% 4.7% **----------------------------QVTEP------LRAMVVGA--PLEDAR-HLAQRYDRIRQEVEVQTAEVARRQLKSKEAG----ATADNAF--**

27 gi134128spP25343.1RV161_YEAST 86.9% 6.0% **----------------GP-------LRETVLDP------ITKFS----TYFKEIE-EAIKKRDHKKQDFDAAKAKVRRLVDKP---AKD-ASKLPRA--E**

28 gi190409230gbEDV12495.1 73.3% 8.4% **-----------------------LELMTTAIEP-----------------YRNVR-----------KNFDFYQGKYDSMLASYQA------IRISKTSLE**

29 gi123888297spQ1LU86.1F92A1_DANRE 81.8% 6.4% **------------------------RLEVKVIEPLK---AYGNIVKTKREDLKQTQ-SARNR---------EAKQMQQLERMRQRN--PSDRQIISQA--E**

30 gi74676202spO94478.1MU137_SCHPO 83.0% 6.8% **----------------YK-------ARVCYLDF------LKRYL----VQAKDFH-SARKKLESRRQAYESLLQKSFKEKKEDSR-----------L--E**

31 gi81880317spQ99N37.1RHG17_RAT 86.9% 8.4% **----------------VF-------VEKEIMDP------LYGIAE---VEIPNIQ-KQRKQLARLVLDWDSVRARWNQAHKSSGTNFQGLPSKIDTL--K**

32 gi730455spP39743.1RV167_YEAST 88.1% 3.5% **----------------AL-------VEEKIVTP------CQELL----KIITYIR-KMATKRNHKKLDLDRHLNTYNKHEKKKEPTAKD-EERLYKA--Q**

33 gi23943858refNP_037453.1 84.1% 6.1% **ELSAIGSDTTPLPSWAALNSSTWGSLKQALKGLSVEFALLADKAAQQGKQEENDVVEKLNLFLDLLQSYKDLCERHEKGVLHKHQRALHKYSLMKRQMMS**

34 gi226520535gbACO66524.1 91.5% 7.2% **-------VRPAC---LEQ-------LGNSVNKP------VGQLCE----EFPAYQ-QCVDKRTHYMLDMDAYERKLQKARQFAKD-----PAKVPHR--E**

35 gi66805481refXP_636473.1 80.1% 4.0% **------------------------ILENYYHDP------LRVYL----SQFRDIR-SRLEELDLRRLDMDRYYRDYNIKANKGKD----ASSLQK---TE**

36 gi66814788refXP_641573.1 86.9% 3.9% **----------------NE-------VQDVFYTP------LNNFVDYDFAEVVESN-KRVTKAKD---DYDQSLGKISASIKKSKQGIDE--GRLFLY--E**

37 gi66811612refXP_639985.1 82.4% 4.8% **------------------------QMAEPYTKP------LSDYI----SQYKEAR-KRTEELTTRKVDMDRYKNEVGKLREKGAG----SSSKAKLAPTE**

38 gi66808477refXP_637961.1 83.5% 4.9% **-----------------------------LINP------VGKFQDTE---IKKAR-DSKHKQDQIRIRYDTALEKL-QEARKKND---ANSLKVKGL--E**

39 gi66815771refXP_641902.1 84.1% 8.3% **-----------------------------VSDP------LKSIIKTE---LKQAK-ESKREYDRVRVAFDAHLSEL-ANLRKQKN---VKPPKIQES--E**

40 gi226457821gbEEH55119.1 89.8% 6.5% **-------VKPLC---LEQ-------LDSAVVAP------VSQLVD----DFPAYA-PCVDKRRAYMLDVDAYERKLDKARTKSKD-----PGMAPHR--E**

41 gi66826123refXP_646416.1 75.0% 4.2% **-------------------------------QP------LSTVADELENFLPLIT-EMRKRVFESHTEYENAVSKLYSLAKNLQP----TEKKMIEA--E**

42 gi66815801refXP_641917.1 86.9% 4.5% **------------------------SISQKWLQQLN---EYG------KSDCKDGQ-LAKNRFDKARLSFDEASEQFKQLRKKQNN--INNEKLL-EA--E**

43 gi115458110refNP_001052655.1 86.4% 6.8% **MEEMGTCLLEKT-----ALNYDDDDSGRVLMMLGKAQFELQKFVDNY--RTNIIN-TITNPSESLLKELQVVEEMKELCDHKRQE----YEAMRAAY--R**

44 gi218202434gbEEC84861.1 86.4% 3.1% **LQEMGTCLLKRV-----TPN-KDGINDKVLLLLGKAQSELRKLVDSY--RVHVLN-TITTPSQSLLNELQTVEEMKHQCDEKREL----FEFLLNAQ--K**

45 gi67468783refXP_650391.1 81.8% 6.1% **------------------------KIKEEVIEP------IQRLV----EACTIME-KRTKTLGQRRLDMDAAHDKYESIAKKAPE----KQNGLAEA--E**

46 gi67467978refXP_650059.1 90.3% 7.8% **-------DIFLP---------------------------LKEYS----QQYKVIE-ERIVELKKRQIDMDHAHNRYGTNVLKNKP-----QHIITNT--K**

47 gi67468969refXP_650476.1 79.5% 4.7% **------------------------RIMNEVVTP------LKEYL----KQFKRID-KKIQECHKRRIDMDRIKENEQQINVM---------TKLEDF--K**

48 gi183234774refXP_656019.2 83.0% 5.2% **------------------------RIESDIIVP------LKTYM----EQFKVME-KRFEICHNRRVDMDRYHDSVLSISKKPPG----KQSGLGEA--Q**

49 gi30685727refNP_180907.2 86.4% 5.2% **LRELGACLLEKT-----ALN-DDEESGRVLIMLGKLQFELQKLVDKY--RSHIFQ-TITIPSESLLNELRIVEEMQRLCDEKRNV----YEGMLTRQ--R**

50 gi30688552refNP_189326.2 84.7% 7.9% **LGEMGSCL-EQI-----APH-NDEESSRILFMLGKVQSELQRLLDTY--RSHIFE-TITSPSEALLKDLRYVEDMKQQCDGKRNV----YEMSLV----K**

51 gi67471335refXP_651619.1 82.4% 6.5% **------------------------IIYCQVIEP------MKIFI----EEWKVYY-KRINIMKNRKIDMDRHFEKLEQIKKKGQK----KQNGKKEA--E**

52 gi66809051refXP_638248.1 81.8% 4.9% **-----------------------------LKGT------IDQFINRD---IKVVR-TSRKNFDKIKSMYESIDGKVNTNANKGKG---INLVKQAEL--Q**

53 gi183233566refXP_655372.2 83.5% 6.2% **------------------------GLMNELATP------LRAYG----QQFKELL-GRCKVAEKRKEDMEFYNERLMEITKKPVN----RQKGLADA--Q**

54 gi118394946refXP_001029831.1 74.4% 6.6% **-------------------------LTNLVISP------LKNYTNVAPDQLKDIK----KKFQKLQEEFEAV--ELKNSQ-QKKATL----QKDKNH--Y**

55 gi195998291refXP_002109014.1 76.7% 4.5% **-EVVDKCVVRL-----------SQNVVA-TVDQ------FNSLFPTA------KK-AIDAHKKSFETY-ENYREKLQKCENSGDG-----SNRYQMT--Q**

56 gi67480227refXP_655464.1 79.0% 5.5% **------------------------EIEQDLIYP------LRQHI----EYYDDLQ-RRIDDCHKTRIDMDRHKEKLESLMKK-TG----KEGKINEY--Q**

57 gi123437348refXP_001309471.1 82.4% 4.9% **-----VSIVELLNSFSETMNTHYDMISTAVISP------LTQFINNDVKNAEQLG----KQAEKMYDNYHSTFEKYVSLSKKKTSDTEIEEFDMKLM--Q**

58 gi66817346refXP_642526.1 77.8% 3.0% **-------------------------VMEGLIKP------IQILIG-AIDEKKQYR----KKFDKAVQEYENIISKIKHQQTQKKIDI----LKIYNY--E**

59 gi66811120refXP_639268.1 84.1% 5.0% **-------------------------ITNRFVLP------AENFVHTVLGPSRDAK----GKFRKSRLEYDSALSKLKIAQQATNIDL----RNLYIC--Q**

60 gi183231363refXP_656153.2 81.2% 4.2% **------------------------KLQKKVMKP------LKMYL----EAVTVLE-KRVKILDERGKAMEKAEHAYHDMLKKPEN----KQVGLSDL--K**

61 gi159480288refXP_001698216.1 86.4% 5.0% **----------------------RQRLDDEVIKP------LRSWLMAY----RTVS-ERMEKLEALRLELDSRRRTVDSLEEKCDKLHKTAPAAKDKDKHE**

62 gi67476764refXP_653934.1 88.6% 9.5% **-------VVISP---------------------------LLELK----KMYNILS-ARHAILKVRHNDCDRFQDQMNNSKGENHV-----Q-----A--K**

63 gi219120544refXP_002181008.1 89.8% 13.5% **-------YAVEWEKVVVTRVGNGLKVVQDLRRD------LDHYQ----KKVEAMR-LSVNQAMSKGKNVKA---DTAERLKRNEE-----KLISAKQ--T**

64 gi154416821refXP_001581432.1 76.1% 4.8% **-----SSLKGILDIINGGFQCHFDTLKNQIIQD------ITNFVNKDFAQLQKFH----DDHKKIHDAYRASQEKYVALSAKDIDKPSSED---PLI--D**

65 gi167376424refXP_001733989.1 90.3% 8.3% **-------DIFLP---------------------------LKEYS----QQYKVIE-ERIVELKKRQIDMDHAHNSYGTNVLKNKP-----QQIITNT--K**

66 gi123447384refXP_001312432.1 80.1% 7.4% **------------------------RIEPLIVSP------LAKFQSEV-MRLVEVR----KQRDEAVKQYDHARANYKYLSEKKSSGF-------EKA--E**

67 gi168036718refXP_001770853.1 79.0% 4.5% **VSDMASYFVAPG-----VLD--DQDIVCVLYIF------FGNCHDKY--WFAIV-------------DLVFKETKKQYDERRQSL----YHHRLRI----**

68 gi123475188refXP_001320773.1 80.1% 7.0% **------------------------LIDQNVVAP------LSVYQKET-DRLKDIQ----KQRKPLRKEYDQARSKLKWLQDHNGKVG-------E-I--E**

69 gi167538764refXP_001751041.1 89.2% 8.3% **-----------------------------VAES------LRQYVARD---VRGVK-DLAKLYHKMSDDLDAARARRAACPPSSSH----LSLSLSPQ--P**

cov pid **301**  **. . . . : . . . . 4** **400**

1 BdpA_Shewanella_genus 100.0% 100.0% **ENYSK--VMSKDEQTRA-QIMAD----VN-------------ANI--SAKADALLAANSNAEL--VQYMIKTTEKMDEF---------Q-EFKERFNQIT**

2 BdpA_Shewanella_oneidensis 100.0% 96.0% **ENYSK--VMSKDEQTRS-EIMAD----VN-------------TNI--SAKAEALLASNSNAEL--VQYMIKTTEKMDEF---------Q-EFKERFNQIT**

3 BdpA_Shewanella_xiamenensis 100.0% 96.6% **ENYSK--VMSKDEQTRA-QIMAE----VN-------------ANI--SAKAEALLASNSNAEL--VQYMIKTTEKMDEF---------Q-EFKERFNQIT**

4 BdpA_Shewanella_decolorationis_S12 100.0% 97.2% **ENYSK--VMSKDEQTRA-QIMAD----VN-------------ANI--SAKAEALLASNSNAEL--VQYMIKTTEKMDEF---------Q-EFKERFNQIT**

5 PmtA_Agrobacterium_tumefaciens 77.8% 4.8% **FDLDATLGEHKGQMFDS------VVSAVPMLNFP--------------MAARIKLLDELLKRVP-HGRPVVQISYGPISPIVAQPHLYHIRHFDFIVRNI**

6 FtsA_Shewanella_oneidensis 98.9% 4.3% **HTLAEV-VEPRYQELFE-LVLKE-------LKDSGLEDQIAAGIVLTGGTASIQGVVDIAEATFGMPVRVASPLPVKGL---------Y-EYVDQSIYST**

7 FtsA_Escherichia_coli 100.0% 3.3% **QTLAEV-IEPRYSELLN-LVNEEILQLQEKLRQQGVKHHLAAGIVLTGGAAQIEGLAACAQRVFHTQVRIGAPLNITGL---------T-DYAQEPYYST**

8 MamY_Magnetospirillum_magneticum 85.2% 8.8% **ATTQLIRLNQEGRALAAPAQASGTDEAMTLLAQL---FREHREAVAAQLEAQASATAQLVQVTR-----DSRDGIVDELR---SQRVLSQEITQELSQIT**

9 gi99031622pdb1X03A 80.7% 6.9% **EELRQA--LEKFDESKE-IAESS----XF-------------NLL----EXDIEQV-SQLSAL-----VQAQLEYHKQA---------V-QILQQVTVR-**

10 gi149241632pdb2ELBA 80.7% 4.8% **VKYEVT--EDVY----------TSRKKQH---------QTXXHYFCALNTLQYKKKIALLEPL--LGYXQAQISFFKXG---------S-ENLNEQLEE-**

11 gi151568102pdb2V0OA 55.1% 4.0% **QKMTET--AQKFQDIEE-----THLIHIKEIIGSL-----------------SNAIKEIHLQIGQV----------------------------------**

12 gi119389482pdb2FICA 84.1% 7.1% **EELIKA--QKVFEEMNV-DLQEE----LP-------------SLW----NSRVGFYVNTFQSI-----AGLEENFHKEM---------S-KLNQNLNDV-**

13 gi14277759pdb1I49A 83.5% 7.8% **ATFQAH--RDKYEKLRG-----DVAIKLKF--------------------LEENKIKVMHKQLL--LFHNAVSAYFAGN---------Q-KQLEQTLQQ-**

14 gi116666967pdb2D1LA 80.1% 3.8% **SALQDV--NDKYLLLEE-------------------------------------TEKQAVRKAL----IEERGRFCTFISXLRPVIEEEISXLGEITH--**

15 gi73536002pdb1ZWWA 80.7% 6.4% **EELRQA--LEKFDESKE-IAESS----MF-------------NLL----EMDIEQV-SQLSAL-----VQAQLEYHKQA---------V-QILQQVTVR-**

16 gi162330107pdb2RAIA 75.6% 6.8% **EKVKES---DKLVATSK-ITLQDKQNXVKRVSIX------SYALQAEXNHFHSNRIYDYNSVIR--LYLEQQVQFYETI---------A-EKLRQALSR-**

17 gi56404535spQ6XZF7.1DNMBP_HUMAN 80.7% 5.6% **EELQSA--RNNYEALNA-QLLDE----LP-------------KFH----QYAQGLFTNCVHGY-----AEAHCDFVHQA-------------LEQLKPLL**

18 gi41018158spQ9Y371.1SHLB1_HUMAN 85.2% 5.3% **QELRIT--QSEFDRQAE-ITRLL----LE-------------GIS----STHAHHL-RCLNDF-----VEAQMTYYAQC---------Y-QYMLDLQKQ-**

19 gi119584059gbEAW63655.1 85.2% 6.0% **EELRPV--REDFEAKNR-QLLEE----MP-------------RFY----GSRLDYFQPSFESL-----IRAQVVYYSEM---------H-KIFGDLSHQ-**

20 gi79512687refNP_196834.3 77.8% 4.3% **VAAALE--QELH----------TSRSMFE---------QARFNLVTALSNVEAKKRFEFLEAV--SGTMDAHLRYFKQG---------Y-ELLHQMEPY-**

21 gi6322148refNP_012223.1 80.7% 8.1% **ASLRVQ--METLEDQFA-QVTEDATVCLQ-------------EVI-----SHANFS-EDLKEL-----AKAQAEYFETS---------A-GLMKEFLSN-**

22 gi11359444pirT49496 77.8% 7.5% **SKYQTK--RKNFELKRF-----DYSSFMQDL-------------------SGGRKEQEILSHLT--KYADAQAKAFLTT---------A-KKVEG-----**

23 gi223461365gbAAI40764.1 82.4% 3.5% **Q-----------EADLQ--VDKERHNFFESSLD----------YVYQIQEVQESKKFNIVEPVL--AFLHSLFISNSLT---------V-ELTQDFLPY-**

24 gi71052100gbAAH51194.2 80.7% 4.5% **E-----------EATGA--LTLTRKCFRHLALD----------YVLQINVLQAKKKFEILDSML--SFMHAQSSFFQQG---------Y-SLLHQLDPY-**

25 gi119370361spQ9ULH1.3ASAP1_HUMAN 83.5% 6.6% **QHGMIRTEITGAEIAEE--MEKERRLFQLQMCE----------YLIKVNEIKTKKGVDLLQNLI--KYYHAQCSFFQDG---------L-KTADKLKQY-**

26 gi148910033gbABR18100.1 64.2% 4.7% **-KLQAA------ESKLA-----DLISTMSALGKEAA---------SAMMAVEAQQQRLTLQRLL--AMVEAERTYHEHLAEILDKLHAQ-----------**

27 gi134128spP25343.1RV161_YEAST 86.9% 6.0% **KELSLA--KDIFENLNN-QLKTE----LP-------------QLV----SLRVPYFDPSFEAL-----IKIQLRFCTDG---------Y-TRLAQIQQY-**

28 gi190409230gbEDV12495.1 73.3% 8.4% **PSSIKSDALQLFEVQKN---------YLKASLD----------LISAISAVKLSLDKFILESMK--VLKSRSIFITKDSGRKIDLSPCINEYLDNY----**

29 gi123888297spQ1LU86.1F92A1_DANRE 81.8% 6.4% **SELQRA-TMDATRTTRQ---LEETIDDFE-----------------------KQKIRDIKKVLG--EFVTVEMAFHAKA---------L-EIYTTAYQH-**

30 gi74676202spO94478.1MU137_SCHPO 83.0% 6.8% **EDIRLA--LYKFEESTE-QVKNR----MI-------------ALK----DVEADQY-QQLTEL-----IVYELNFFKES---------T-GILNTIFNS-**

31 gi81880317spQ99N37.1RHG17_RAT 86.9% 8.4% **EEMDEA--GNKVEQCKD-QLAAD----MY-------------NFM----AKEGEYG-KFFVTL-----LEAQADYHRKA---------L-AVLEKALPE-**

32 gi730455spP39743.1RV167_YEAST 88.1% 3.5% **AQVEVA--QQEYDYYND-LLKTQ----LP-------------ILF----SLEAEFVKPLFVSF-----YFMQLNIFYTL---------Y-NRLQDMKIP-**

33 gi23943858refNP_037453.1 84.1% 6.1% **ATAQNR-EPESVEQLES--RIVEQENAIQTMELRNYFSLYCLHQETQLIHVYLPLTSHILRAFV-----NSQIQGHKEMS----------KVWNDLRPK-**

34 gi226520535gbACO66524.1 91.5% 7.2% **EQFTKA--QRRYTYFSD-KLVED----LT-------------LLD----ANRYELAGFLIEGF-----VEMQEFQSQRQ---------K-DVLAGLAQGK**

35 gi66805481refXP_636473.1 80.1% 4.0% **NKHTKT--KEAY----Q-ELSDEIMKDMY-----------------ALFDDRKLAFDPSFACFI-----NRNNEYFARA---------A-AEYQGALASV**

36 gi66814788refXP_641573.1 86.9% 3.9% **QELEKL--KEQYEGSNK-DLEIK----LA-------------ELE----HKNETKYLKSLILF-----INSQYHFYNRA---------S-KLFANLKPKL**

37 gi66811612refXP_639985.1 82.4% 4.8% **EKLRIC--KEGY----D-SLHEELLNDLP-----------------RLNEDKLIFIDYLVASLI-----KYQSEFHKKV---------S-YEWSPLPSLT**

38 gi66808477refXP_637961.1 83.5% 4.9% **NECNEI--KVEYDAVTA-----EFTQTMD--------------------NLNQEMNKQLVEELR--EYTLQQLAFYKQA---------A-ALWEETSDLL**

39 gi66815771refXP_641902.1 84.1% 8.3% **EECERL--RTNFERVGI-----DTTCLLR--------------------DTNVITEFETVEKLC--DYLDSYHTFFQKG---------YRWLAQMIPDIY**

40 gi226457821gbEEH55119.1 89.8% 6.5% **EQYARA--QRRFAHFSD-KLVED----LT-------------LLD----ANRFELAAFLLEGF-----IETQDFMMQRT---------R-DV-----HSV**

41 gi66826123refXP_646416.1 75.0% 4.2% **KEVASL---------KK--VLDKTLFEFDAIFKEGI-------------SIQTKSLKLFYACMK------AQQEYYERG---------L-QRFQAMKPS-**

42 gi66815801refXP_641917.1 86.9% 4.5% **EDLDYA-TQQFSDIASE---SLQTMDDII-----------------------VKHNLDSFESAS--STIQSYKDFFQKG---------L-DHCLSVQSDL**

43 gi115458110refNP_001052655.1 86.4% 6.8% **EKGRSR--HSKTETLSS-EQLQAYFLDYQ---------EDAALFIFRLKSLKQGQFRSILTQA--ARHHSAQLSFFRRG---------L-KYLEALEPH-**

44 gi218202434gbEEC84861.1 86.4% 3.1% **EKGRSK--NAKSDIGAS-EQLKQAQDDYQ---------EEATLFLFRLKSLKQGQFRSLFTQA--ARHHAAQLNLFRKG---------L-KSLEAVEPH-**

45 gi67468783refXP_650391.1 81.8% 6.1% **TRYNEA--KDNY----D-YLRNEIVTDIN-----------------KCIEQVKIQFPQICVNCM-----KSYTDYINEL---------N-EIWTKVPNII**

46 gi67467978refXP_650059.1 90.3% 7.8% **TKYEQA--RDFYFYLRN-EIIDD----MK-------------KLN----NNYIKIVEPFFVKL--FQNDILIRDIYNES---------L-DSIEKLLPS-**

47 gi67468969refXP_650476.1 79.5% 4.7% **NKYEKL--RDELVFDLN-HLLNNIDNSIE-----------------QMTTIMTTIFEDFYSKQY-----FNFSIFNNQI---------K-YQ--------**

48 gi183234774refXP_656019.2 83.0% 5.2% **NKYNVA--RDLY----N-YLRNEIIADVE-----------------KLTSSAEEVVSPICGTLI-----VSYTDYLNHL---------N-NYWGEASEVS**

49 gi30685727refNP_180907.2 86.4% 5.2% **EKGRSK--GGKGETFSP-QQLQEAHDDYE---------NETTLFVFRLKSLKQGQTRSLLTQA--ARHHAAQLCFFKKA---------L-SSLEEVDPH-**

50 gi30688552refNP_189326.2 84.7% 7.9% **EKGRPK--SSKGERHIP-PESRPAYSEFH---------DEATMCIFRLKSLKEGQARSLLIQA--VRHHTAQMRLFHTG---------L-KSLEAVERH-**

51 gi67471335refXP_651619.1 82.4% 6.5% **AKYKAS--KETY----I-LIRNEIINDIS-----------------KLNERINEINEKIESSII-----LGFTEYINCL---------N-LSWDKVPEML**

52 gi66809051refXP_638248.1 81.8% 4.9% **QERDFL--RGRLNQVGQ-----ESLSTIK--------------------LANESNSVEIMEQMV--EYMEN----MQQA---------VKALSSQMNQLQ**

53 gi183233566refXP_655372.2 83.5% 6.2% **TDFTYA--KEKY----E-WLKQELIEDTN-----------------KLCKDFQGVVLPVVRALM-----VNFTEVMNGM---------N-GIWEEVPKKV**

54 gi118394946refXP_001029831.1 74.4% 6.6% **SNISKNHLQVA-KQYE--QIRYEYTCNLN--------------------KLQYEMNTEISNSIC--ALLQTFAVI-----------------FQQIKQAI**

55 gi195998291refXP_002109014.1 76.7% 4.5% **VKLQSA--SKEFAMEDN-----KLASIMSSM-------------------LEYRV-QKLGAAFL--SLIDIESKAFQTT---------A-KAHKA-----**

56 gi67480227refXP_655464.1 79.0% 5.5% **IKFDAE--KERY----S-KLRSDIINETE-----------------CLEEERMTIARPIMSSLL-----VCYTKYLNSV---------S-KGWKQIEKVM**

57 gi123437348refXP_001309471.1 82.4% 4.9% **SHGRAV--FADFQLSRQ---LELVERKKNV---------------------------EVLTEFI--AFLNMLGTTYEQC--ADFFKGAKD-HFNTIRQA-**

58 gi66817346refXP_642526.1 77.8% 3.0% **KEKSK----LK-QNYE--NVKNEYIYYLT--------------------DTENRMHTEFLDLLV--LHYESMQLLNGNA---------Y-AEYAGIKTYI**

59 gi66811120refXP_639268.1 84.1% 5.0% **SHFATYKLRFAHRQYEA-QVRLADTVQRH--------------------GFEYLVQS------I--NLLATLHDLYSNS---------C-THLNENEQSI**

60 gi183231363refXP_656153.2 81.2% 4.2% **VNYTNA--RDSW----E-YLRDELSVDIK-----------------KVLDEITSNFGQICSGFM-----KTYSKYMKNT---------Q-EVWDKLHDY-**

61 gi159480288refXP_001698216.1 86.4% 5.0% **QEMEKM-SQLLLHKQDKCNRTKNAFEELEKMVYNSL---NTLVKDTGVLRDYTGLSLSILQDCF----QRGHSAFSTATP-------LLDYNSTSDNHMY**

62 gi67476764refXP_653934.1 88.6% 9.5% **QKYERS--LDLYNYLRS-ELVED----MK-------------NII----STSMLFYNETMIGL--IGSDIYEDEQITET---------R-SSLFNLQNSM**

63 gi219120544refXP_002181008.1 89.8% 13.5% **FNKSATDLCILMEEVTE-RSWRD----LH-------------PLLLKCAQFDMTLASDESSILSGLNAVVSALKEV------------------------**

64 gi154416821refXP_001581432.1 76.1% 4.8% **AHAQNA--ISYFDYTSK---MENIELQFQILIP------------------------SILTNFI--SSV---------CDPMKFVLGSIDCSKSSIDKC-**

65 gi167376424refXP_001733989.1 90.3% 8.3% **TKYEQA--RDFYFYLRN-EIIDD----MK-------------KLN----NNYIKIVEPFFVKL--FQNDILIRDIYNES---------L-ESIEKLLPS-**

66 gi123447384refXP_001312432.1 80.1% 7.4% **AEYKLT--KENYEKY-----NEDFIETVK-----------------KLMNQRENGL-DTPAKTL----IAILSQFLMQL---------F-REAQKFRTTF**

67 gi168036718refXP_001770853.1 79.0% 4.5% **AKGRSK--IGKTDAQEE-EQLENVREQFE---------EVSQFLGDRLLSLRQGRPRSLITQT--ARHHAAQMQLFSKV---------L-TSLHGIEPHM**

68 gi123475188refXP_001320773.1 80.1% 7.0% **AQNQKT--QEAYNKYSV--LNNDFIQGVN-----------------RLVMQRAQYL-ETPFRNF----VGIFSKFMCSV---------T-NEMERVKTS-**

69 gi167538764refXP_001751041.1 89.2% 8.3% **ENCRLT--KEGYLFKRAHNVMRTWSRRWF---------ILRGGQLLYVNRDKEEPPQAFVEDLR-ICTVKAEPSESIDR---------T-NCFEIITPS-**

cov pid **401**  **.]** **411**

1 BdpA_Shewanella_genus 100.0% 100.0% **S----------**

2 BdpA_Shewanella_oneidensis 100.0% 96.0% **S----------**

3 BdpA_Shewanella_xiamenensis 100.0% 96.6% **S----------**

4 BdpA_Shewanella_decolorationis_S12 100.0% 97.2% **S----------**

5 PmtA_Agrobacterium_tumefaciens 77.8% 4.8% **PPAQLWTYTRA**

6 FtsA_Shewanella_oneidensis 98.9% 4.3% **GVGLLHY----**

7 FtsA_Escherichia_coli 100.0% 3.3% **AVGLLHY----**

8 MamY_Magnetospirillum_magneticum 85.2% 8.8% **QSRTV------**

9 gi99031622pdb1X03A 80.7% 6.9% **-----------**

10 gi149241632pdb2ELBA 80.7% 4.8% **-----------**

11 gi151568102pdb2V0OA 55.1% 4.0% **-----------**

12 gi119389482pdb2FICA 84.1% 7.1% **-----------**

13 gi14277759pdb1I49A 83.5% 7.8% **-----------**

14 gi116666967pdb2D1LA 80.1% 3.8% **-----------**

15 gi73536002pdb1ZWWA 80.7% 6.4% **-----------**

16 gi162330107pdb2RAIA 75.6% 6.8% **-----------**

17 gi56404535spQ6XZF7.1DNMBP_HUMAN 80.7% 5.6% **SL---------**

18 gi41018158spQ9Y371.1SHLB1_HUMAN 85.2% 5.3% **-----------**

19 gi119584059gbEAW63655.1 85.2% 6.0% **-----------**

20 gi79512687refNP_196834.3 77.8% 4.3% **-----------**

21 gi6322148refNP_012223.1 80.7% 8.1% **-----------**

22 gi11359444pirT49496 77.8% 7.5% **LLPQ-------**

23 gi223461365gbAAI40764.1 82.4% 3.5% **-----------**

24 gi71052100gbAAH51194.2 80.7% 4.5% **-----------**

25 gi119370361spQ9ULH1.3ASAP1_HUMAN 83.5% 6.6% **-----------**

26 gi148910033gbABR18100.1 64.2% 4.7% **-----------**

27 gi134128spP25343.1RV161_YEAST 86.9% 6.0% **-----------**

28 gi190409230gbEDV12495.1 73.3% 8.4% **-----------**

29 gi123888297spQ1LU86.1F92A1_DANRE 81.8% 6.4% **-----------**

30 gi74676202spO94478.1MU137_SCHPO 83.0% 6.8% **-----------**

31 gi81880317spQ99N37.1RHG17_RAT 86.9% 8.4% **-----------**

32 gi730455spP39743.1RV167_YEAST 88.1% 3.5% **-----------**

33 gi23943858refNP_037453.1 84.1% 6.1% **-----------**

34 gi226520535gbACO66524.1 91.5% 7.2% **LPPK-------**

35 gi66805481refXP_636473.1 80.1% 4.0% **QHINEF-----**

36 gi66814788refXP_641573.1 86.9% 3.9% **DNIEKY-----**

37 gi66811612refXP_639985.1 82.4% 4.8% **KHVDEY-----**

38 gi66808477refXP_637961.1 83.5% 4.9% **SS---------**

39 gi66815771refXP_641902.1 84.1% 8.3% **EY---------**

40 gi226457821gbEEH55119.1 89.8% 6.5% **IVKK-------**

41 gi66826123refXP_646416.1 75.0% 4.2% **-----------**

42 gi66815801refXP_641917.1 86.9% 4.5% **EIQ--------**

43 gi115458110refNP_001052655.1 86.4% 6.8% **-----------**

44 gi218202434gbEEC84861.1 86.4% 3.1% **-----------**

45 gi67468783refXP_650391.1 81.8% 6.1% **SSIPEVDLKQE**

46 gi67467978refXP_650059.1 90.3% 7.8% **-----------**

47 gi67468969refXP_650476.1 79.5% 4.7% **-----------**

48 gi183234774refXP_656019.2 83.0% 5.2% **SNFRICALDPA**

49 gi30685727refNP_180907.2 86.4% 5.2% **-----------**

50 gi30688552refNP_189326.2 84.7% 7.9% **-----------**

51 gi67471335refXP_651619.1 82.4% 6.5% **RNMNTGDINTQ**

52 gi66809051refXP_638248.1 81.8% 4.9% **VP---------**

53 gi183233566refXP_655372.2 83.5% 6.2% **EN-----LPSN**

54 gi118394946refXP_001029831.1 74.4% 6.6% **QRNQINIQ---**

55 gi195998291refXP_002109014.1 76.7% 4.5% **LDNH-------**

56 gi67480227refXP_655464.1 79.0% 5.5% **K-----EIPPN**

57 gi123437348refXP_001309471.1 82.4% 4.9% **-----------**

58 gi66817346refXP_642526.1 77.8% 3.0% **--DSLRTW---**

59 gi66811120refXP_639268.1 84.1% 5.0% **K--EIKDY---**

60 gi183231363refXP_656153.2 81.2% 4.2% **-----------**

61 gi159480288refXP_001698216.1 86.4% 5.0% **NH---------**

62 gi67476764refXP_653934.1 88.6% 9.5% **EKT--------**

63 gi219120544refXP_002181008.1 89.8% 13.5% **-----------**

64 gi154416821refXP_001581432.1 76.1% 4.8% **-----------**

65 gi167376424refXP_001733989.1 90.3% 8.3% **-----------**

66 gi123447384refXP_001312432.1 80.1% 7.4% **PEYVLNG----**

67 gi168036718refXP_001770853.1 79.0% 4.5% **KQVTKE-----**

68 gi123475188refXP_001320773.1 80.1% 7.0% **-----------**

69 gi167538764refXP_001751041.1 89.2% 8.3% **-----------**
